# EEG Signal Processing Techniques and Applications

**DOI:** 10.3390/s23229056

**Published:** 2023-11-09

**Authors:** Yifan Zhao, Fei He, Yuzhu Guo

**Affiliations:** 1 School of Aerospace, Transport and Manufacturing, Cranfield University, Cranfield MK43 0AL, UK; 2 Research Centre for Computational Science and Mathematical Modelling, Coventry University, Coventry CV1 5FB, UK; fei.he@coventry.ac.uk; 3 School of Automation Science and Electrical Engineering, Beihang University, Beijing 100191, China; yuzhuguo@buaa.edu.cn

## 1. Background

Electroencephalography (EEG) is a widely recognised non-invasive method for capturing brain electrophysiological activity. It stands out for its cost-effectiveness, portability, ease of administration, and widespread availability in most hospital settings. Unlike other neuroimaging modalities focused on anatomical structure, such as MRI, CT, and fMRI, EEG excels in providing ultra-high time resolution, a crucial asset for in-depth insights into brain functioning [1].

The empirical interpretation of EEG data predominantly relies on the identification of abnormal frequency patterns in distinct biological states (e.g., wakefulness versus sleep [2]) and the spatial-temporal and morphological characteristics of paroxysmal [3] and persistent discharges [4]. Reactivity to external stimuli and activation procedures, such as intermittent photic stimulation or hyperventilation, also plays a significant role in EEG analysis [5,6]. While these practical approaches are valuable in many cases, they often fall short of capturing the intricate, dynamic, and nonlinear interactions among various anatomical constituents of the brain networks. These interactions frequently remain hidden within the EEG recordings, surpassing the observational capabilities of even highly trained physicians in the field. This oversight is supported by substantial evidence across various neurological conditions, including epilepsy, neurodegenerative dementias, neuropsychiatric and movement disorders, as well as normal cognitive paradigms [7]. 

Moreover, EEG data are inherently nonstationary and susceptible to various sources of noise, notably frequency interference. Consequently, the effective removal of noise from raw EEG data is imperative to extract meaningful information that accurately reflects brain activity and states [8]. In recent years, approaches based on machine learning have attracted considerable attention due to their exceptional capability to unveil underlying patterns within noisy EEG recordings for various applications. 

This Special Issue serves as a platform for the dissemination of original high-quality research in EEG signal pre-processing, modelling, analysis, and their applications, with a particular focus on the utilisation of machine learning and deep learning techniques. The range of applications covered includes the following:

- Healthcare applications, including epilepsy (contributions 1–3) and anaesthesia (contribution 4);
- Studies related to emotion (contributions 5–7);
- Research on motor imagery (contributions 8–10);
- Investigations into external stimulations (contributions 11–13);
- Research concerning mental workload (contributions 14–15);
- Studies in satisfaction (contribution 16).

## 2. Overview of Contributions

Alreshidi et al. (contribution 14) reported a novel multimodal approach for mental state detection in pilots using EEG signals. The innovative nature of this study lies in its combination of advanced automated preprocessing techniques, Riemannian geometry-based feature extraction, and ensemble learning models, which, together, provide a detailed and accurate characterization of pilot mental states, ultimately leading to a safer and more efficient aviation system.

Borra et al. (contribution 8) investigated the power and connectivity in the Alpha and Beta bands of EEG recordings during planning goal-directed movement. It was suggested that alpha and beta oscillations are functionally involved in the preparation of reaching in different ways, with the former mediating the inhibition of the ipsilateral sensorimotor areas and disinhibition of visual areas, and the latter coordinating disinhibition of the contralateral sensorimotor and visuomotor areas. This study contributes to enriching the description of the neural mechanisms underlying reaching movement preparation in healthy subjects, leading to a better comprehension of the neurophysiological correlates.

Mockevičius et al. (contribution 12) produced a methodology for determining the individual gamma frequency from EEG data where subjects received auditory stimulation consisting of clicks with varying inter-click periods. This work demonstrates that the estimation of individual gamma frequency is possible using a limited number of both the gel and dry electrodes from responses to click-based chirp-modulated sounds.

Oikonomou et al. (contribution 13) proposed a novel framework to recognise the cognitive and affective processes of the brain during neuromarketing-based stimuli using EEG signals. More specifically, an extension of the basic Sparse Representation Classification (SRC) scheme was proposed that utilises the graph properties of neuroimaging data. The experimental analysis provides evidence that EEG signals could be used for predicting consumers’ preferences in neuromarketing scenarios.

Yang et al. (contribution 2) presented novel EEG–EEG or EEG–ECG transfer learning strategies to explore their effectiveness for the training of simple cross-domain convolutional neural networks (CNNs) used in seizure prediction and sleep staging systems, respectively. It was concluded that transfer learning from an EEG model to produce personalised models for a more convenient signal can both reduce the training time and increase the accuracy; moreover, challenges such as data insufficiency, variability, and inefficiency can be effectively overcome.

Abdel-Hamid (contribution 5) introduced a subject-dependent emotional valence recognition method using EEG recordings. Time and frequency features were computed from only two channels and state-of-the-art performance was achieved and validated by a benchmark DEAP dataset. This approach would thus be highly attractive for practical EEG-based emotion AI systems relying on wearable EEG devices.

Shi et al. (contribution 4) proposed a deep residual shrinkage network to estimate the depth of anesthesia (DoA) from EEG signals. The proposed procedure is not merely feasible for estimating DoA by mimicking patient state index (PSI) values but also inspired us to develop a precise DoA-estimation system with more convincing assessments of anesthetisation levels.

Yuvaraj et al. (contribution 6) contributed another emotion recognition approach that uses features including statistical features, fractal dimension (FD), Hjorth parameters, higher order spectra (HOS), and those derived using wavelet analysis. The results of this research may lead to the possible development of an online feature extraction framework, thereby enabling the development of an EEG-based emotion recognition system in real time.

Kim et al. (contribution 16) reported a study to use EEG measures to reflect user satisfaction in controlling a robot hand. For the moment that dominated satisfaction, it was observed that brain activity exhibited significant differences in satisfaction not immediately after feeding an input but during the later stage. The other indicators exhibited independently significant patterns in event-related spectral perturbations. The results reveal that regardless of subjective satisfaction, objective performance evaluation might more fully reflect user satisfaction.

As an effort in neuromarketing, Shah et al. (contribution 7) proposed an ensemble model for predicting emotion using EEG signals to evaluate the consumer’s opinion toward a product. Automated features were extracted by using a long short-term memory network (LSTM) and then concatenated with handcrafted features such as power spectral density (PSD) and discrete wavelet transform (DWT) to create a complete feature set. This research demonstrates that brain-imaging techniques and tools can help marketers and advertisement agencies to improve their marketing campaigns before launching the product in the market and also during the in-market inspection of the campaign’s success after the launch.

Jochumsen et al. (contribution 10) implemented three performance accommodation mechanisms (PAMs) in an online motor imagery-based EEG to aid people and evaluate their perceived control and frustration for stroke rehabilitation. Within the different types of PAMs, game developers can exercise tremendous artistic freedom to create engaging interactions for Brain–Computer Interface (BCI) training that either directly manipulates the outcomes of a single action or its effect in a bigger task context.

Hu et al. (contribution 9) proposed a novel circulant singular spectrum analysis embedded common spatial pattern method for learning the optimal time–frequency–spatial features to improve the motor imagery (MI) classification accuracy using EEG data. The results confirm that it is a promising method for improving the performance of MI-based BCIs.

Li and Iramina (contribution 11) estimated dynamic functional connectivity between the visual cortex and all the other areas of the brain to find which of them were influenced by visual stimuli. They found that seeing manipulable objects and seeing tools caused similar phenomena in both time and space. There is no evidence suggesting that seeing a manipulable object led to a similar mu rhythm change to seeing an interaction with the same object.

Cao et al. (contribution 15) introduced a sensor fusion method to evaluate cognitive workload based on EEG and functional near-infrared spectroscopy (fNIRS). They explored the classification performance of the features of bivariate functional brain connectivity in the time and frequency domains of delta, theta, and alpha bands, with the assistance of the fNIRS oxyhemoglobin and deoxyhemoglobin indicators. 

Najafi et al. (contribution 1) explored the potential of diagnosing focal and generalised epilepsy using EEG by extracting features from discrete wavelet transform and combining them with an RNN-LSTM classifier. The results show that the theta frequency band was more successful than alpha and beta in the detection procedure.

Alharthi et al. (contribution 3) presented another study on epileptic disorder detection using EEG. The proposed system uses a wavelet decomposition technique and a simple one-dimensional convolutional neural network, along with bidirectional long-short-term memory and attention, to receive EEG signals as input data, pass them to various layers, and finally make a decision via a dense layer. This model can assist neurophysiologists in detecting seizures and significantly decrease the burden, while also increasing the efficiency.
